# The impact of six-week dihydrogen-pyrroloquinoline quinone supplementation on mitochondrial biomarkers, brain metabolism, and cognition in elderly individuals with mild cognitive impairment: a randomized controlled trial

**DOI:** 10.1016/j.jnha.2024.100287

**Published:** 2024-06-21

**Authors:** Sonja Baltic, David Nedeljkovic, Nikola Todorovic, Marijana Ranisavljev, Darinka Korovljev, Jelena Cvejic, Jelena Ostojic, Tyler W. LeBaron, Judi Timmcke, Valdemar Stajer, Sergej M. Ostojic

**Affiliations:** a Applied Bioenergetics Lab, Faculty of Sport and PE, University of Novi Sad, Novi Sad, Serbia; b Molecular Hydrogen Institute Cedar City, UT, USA; c Southern Utah University, Cedar City, UT, USA; d CalerieLife, Irvine, CA, USA; e Department of Nutrition and Public Health, University of Agder, Kristiansand, Norway; f Faculty of Health Sciences, University of Pecs, Pecs, Hungary

**Keywords:** Dihydrogen, Pyrroloquinoline quinone, Brain metabolism, Cognition, Cerebral oxygenation

## Abstract

**Objectives:**

To assess the impact of medium-term supplementation with dihydrogen and pyrroloquinoline quinone (PQQ) on mitochondrial biomarkers, brain metabolism, and cognition in elderly individuals diagnosed with mild cognitive impairment.

**Design:**

A parallel-group, randomized, placebo-controlled, double-blind experimental design, maintaining a 1:1 allocation ratio between the experimental group (receiving the dihydrogen-producing minerals and PQQ) and the control group (receiving the placebo) throughout the trial.

**Setting and participants:**

Thirty-four elderly individuals with mild cognitive impairment (mean age 71.9 ± 3.8 years; 28 females) voluntarily provided written consent to participate in this trial. Participants were assigned in a double-blind parallel-group design to receive either a dihydrogen-PQQ mixture (Alpha Hope®, CalerieLife, Irvine, CA) or placebo twice daily for a 6-week intervention period.

**Methods:**

The primary endpoint was the change in serum brain-derived neurotrophic factor (BDNF) from baseline to the 6-week follow-up; secondary outcomes included cognitive function indices, specific metabolites in brain tissue, brain oxygenation, and the prevalence and severity of side effects. Interaction effects (time vs. intervention) were evaluated using two-way ANOVA with repeated measures and Friedman’s 2-way ANOVA by ranks, for normally distributed data with homogeneous variances and non-homogeneous variances, respectively.

**Results:**

Dihydrogen-PQQ resulted in a significant elevation in serum BDNF levels at the six-week follow-up (*P* = 0.01); conversely, no changes in BDNF levels were observed in the placebo group throughout the study duration (*P* = 0.27). A non-significant trend in the impact of interventions on BDNF levels was observed (treatment vs. time interaction, *P* = 0.14), suggesting a tendency for dihydrogen-PQQ to upregulate BDNF levels compared to the placebo. A significant interaction effect was observed for the Alzheimer's Disease Assessment Scale-Cognitive subscale (ADAS-Cog) scores in the orientation domain (*P* = 0.03), indicating the superiority of dihydrogen-PQQ over placebo in enhancing this cognitive aspect. Cerebral oxygenation saturation exhibited a significant increase following the administration of the dihydrogen-PQQ mixture, from 48.4 ± 7.2% at baseline to 52.8 ± 6.6% at 6-week post-administration (*P* = 0.005). In addition, brain *N*-acetyl aspartate levels significantly increased at seven out of thirteen locations post-intervention in participants receiving the mixture (*P* ≤ 0.05).

**Conclusions:**

Despite the limited number of participants included in the study for interpreting clinical parameters, the dihydrogen-PQQ mixture blend shows promise as a potential dietary intervention for enhancing mental orientation and brain metabolism in individuals with age-related mild cognitive decline.

## 1 Introduction

Mild cognitive impairment (MCI) refers to a condition where an individual experiences cognitive changes greater than what might be expected for their age but doesn't meet the criteria for dementia [1]. Its prevalence varies, estimated at 10%–20% among individuals over 65 years old, although these numbers may fluctuate depending on the studied population and diagnostic criteria [2]. The impact of MCI is substantial, increasing the likelihood of developing dementia, particularly Alzheimer's disease (AD), vascular dementia, or other neurological conditions. Additionally, individuals with MCI often encounter challenges in daily activities and cognitive tasks, resulting in diminished quality of life and independence [3]. MCI is regarded as the transitional stage between healthy aging and dementia, serving as a pivotal target for pharmacological and non-pharmacological interventions intended to delay or impede the progression toward dementia. There is ongoing research exploring various nutritional interventions for MCI (for a detailed review see Ref [4]. and [5]). Among other nutritional compounds, molecular hydrogen (dihydrogen, H_2_) and pyrroloquinoline quinone (PQQ) are suggested to activate metabolic pathways involved in cognition. A recent *in vitro* study demonstrated that a mixture of hydrogen-producing minerals and PQQ increased the levels of peroxisome proliferator-activated receptor-gamma coactivator 1-alpha (PGC-1α) and nuclear factor erythroid 2-related factor 2 (Nrf2), master regulators of cellular senescence, mitochondrial regeneration, and redox homeostasis [6]. Given the documented mitochondrial disturbances associated with MCI [7], the potential use of dihydrogen and PQQ to restore mitochondrial viability and redox homeostasis could offer a safe and effective nutritional strategy in this prevalent condition. However, to date, no human studies have assessed the efficacy of this nutritional mixture in the clinical context of MCI. Therefore, the principal objective of this randomized controlled trial was to evaluate the effects of medium-term supplementation with dihydrogen and PQQ on mitochondrial biomarkers, brain metabolism, and cognition in elderly individuals diagnosed with MCI.

## 2 Methods

### 2.1 Trial design

The study employed a parallel-group, randomized, placebo-controlled, double-blind experimental design, maintaining a 1:1 allocation ratio between the experimental group (receiving the dihydrogen-producing minerals and PQQ) and the control group (receiving the placebo) throughout the trial. No changes were made to the trial design after its initiation. The study is registered at ClinicalTrials.gov (NCT05910047).

### 2.2 Participants

The eligibility criteria for participant inclusion in the trial encompassed several aspects: individuals ≥65 years, mild cognitive impairment (*e.g.*, MMSE scores ≤ 28 points, see below), and informed consent signed. The exclusion criteria comprised severe chronic diseases and acute injuries, the use of dietary supplements within four weeks preceding the study, regular participation in exercise, lack of consent for randomization, and involvement in other concurrent studies. Eligible participants provided voluntary informed written consent, and ethical clearance was obtained from the local Institutional Review Board (IRB) at the University of Novi Sad (#A13-CH-2023). The study adhered to the Declaration of Helsinki (7th revision) and the International Conference of Harmonization Efficacy Guidelines E6. Data collection took place at the Applied Bioenergetics Lab at the University of Novi Sad between September 2023 and February 2024.

### 2.3 Interventions

The experimental group was administered a daily dosage of dihydrogen-PQQ mixture, containing PQQ (20 mg) and dihydrogen-producing elemental magnesium (80 mg). The control group (placebo) received an equivalent amount of non-dihydrogen producing magnesium bicarbonate with organic acids. Participants were directed to take the intervention twice daily, prior to breakfast and dinner, by dissolving the experimental or control tablet in 250 mL of lukewarm water and consuming it immediately. Both interventions were indistinguishable in appearance, texture, and sensory characteristics. The dihydrogen-PQQ mixture (Alpha Hope®) and placebo used in the study were provided by CalerieLife (Irvine, CA). The intervention spanned 6 weeks, during which participants were asked to refrain from using any other nutritional supplements. All participants were also asked to refrain from major changes in diet and physical activity during the study.

### 2.4 Outcomes

A predetermined list of primary and secondary outcome measures encompassed biochemical markers (including safety panel), cognitive function indices, and the prevalence and severity of side effects. In a subset of participants receiving experimental intervention, levels of specific metabolites in brain tissue were evaluated in distinct brain regions, alongside an assessment of brain oxygenation in the prefrontal cortex. All assessments were conducted at two time points: baseline (pre-administration) and the 6-week follow-up (post-administration). The primary endpoint was the change in serum brain-derived neurotrophic factor (BDNF) from baseline to the 6-week follow-up. At each visit to the lab, the participants provided fasting blood samples for biochemical analyses. Blood samples were collected from the antecubital vein using a gel vacutainer, with the samples centrifuged within 10 min at 3,000 *g* to isolate the serum. The serum was frozen at –80 °C and analyzed for biochemical markers and safety panel after the completion of the study. Serum levels of brain-derived neurotrophic factor (BDNF) and irisin were determined with commercial ELISA kits (Abcam Ltd, Cambridge, UK for BDNF, code: AB212166; and Cusabio, Houston, TX for irisin, code: CSB-EQ027943HU). The safety panel indices comprising clinical enzymes, lipid panel and glycemia were analyzed by standard enzymatic methods with an automated analyzer (Hitachi 912, Tokyo, Japan). General cognitive function was evaluated with Mini-Mental State Examination (MMSE), a quick screening tool that helps assessing cognitive impairment; the maximum score a person can achieve is 30 points, with higher scores indicating better cognitive function [8]. The Alzheimer's Disease Assessment Scale-Cognitive subscale (ADAS-Cog) was used to determine twelve cognition subdomains that include both subject-completed tests and observer-based assessments, such as memory, praxis, and language deficiencies. The total score ranges from 0 to 70 points, with higher scores indicating more severe cognitive impairment [9]. The cerebral oxygenation levels (SbO2) and hemoglobin index (tHb) in the prefrontal cortex were tracked using a 4-optode (wavelength range of 680–800 nm) functional near-infrared spectroscopy sensor (Fortiori Design LLC, Hutchinson, MN). Brain levels of total choline (tCho), total creatine (tCr), and *N*-acetyl aspartate (NAA) were determined using 1.5 T proton magnetic resonance spectroscopy (Avanto Scanner, Siemens, Erlangen, Germany). Metabolite spectra were collected from frontal, precentral, and parietal white and grey matter, and thalamus, and processed as previously described [10]. Participants were also asked to report any negative side effects (*e.g.*, gut disturbances, belching, headache) and positive effects (*e.g*., less fatigue, improved sleep) resulting from either intervention throughout the study, using an open-ended questionnaire. No alterations to the trial outcomes were made after the trial commenced. All measurements were performed between 07:00 and 10:00 following an overnight fasting period, and participants refrained from consuming caffeine within the preceding 12 h.

### 2.5 Sample size

The minimal sample size (*n* 34) was determined via power analysis (utilizing G*Power 3.1.9.3, Heinrich-Heine-Universität Düsseldorf) based on an effect size of 0.25 (considered a small effect), an alpha error probability of 0.05, and a power of 0.80. This calculation was designed for two groups and two measurements of study outcomes. Termination criteria involved severe adverse events linked to the intervention or substantial health status changes from other causes. The computer program generated the random allocation sequence, and an independent individual not involved in the study assigned participants to their respective interventions (experimental and control group). Throughout the study duration, both participants and investigators remained unaware of the treatment assignments.

### 2.6 Statistical analyses

The data initially underwent the Shapiro–Wilk test to assess normal distribution and Bartlett's test to examine variance homogeneity. Baseline characteristics between and within groups during the trial were compared using *t* tests for normally distributed data and the Wilcoxon Signed-Ranks Test for matched data, as well as the Mann–Whitney U test for unmatched data for variables not following a normal distribution, and Chi-square test with Yates’s correction for categorical variables. For normally distributed data with homogeneous variances, interaction effects (time vs. intervention) were assessed using two-way ANOVA with repeated measures. When non-homogeneous variances were detected, comparisons were made utilizing Friedman’s 2-way ANOVA by ranks. Post-hoc LSD test and Wilcoxon test were respectively employed for identifying differences between individual sample pairs in 2-way ANOVA and Friedman’s test. A significance level of *P* ≤ 0.05 was set. Any missing data were excluded from the analyses. All statistical analyses were conducted using SPSS version 24.0 for Mac (IBM SPSS Statistics, Chicago, IL).

## 3 Results

The overall count of participants who were randomly assigned, received the designated treatment, and were analyzed for the primary outcome was thirty-four (*n* 34), eighteen in the experimental group and sixteen in the control group. The baseline demographic and clinical characteristics for each group were outlined in Table 1. No significant differences were observed in baseline characteristics between the groups (*P* >  0.05).

**Table 1:** Baseline demographic and clinical characteristics of the study participants.

	Experimental group (*n* = 18)	Control group (*n* = 16)	P*
Age, years, mean ± SD	72.4 ± 3.7	71.4 ± 4.0	0.45

Female, *n* (%)	16 (88.9)	12 (75.0)	0.54

Weight, kg, mean ± SD	68.3 ± 12.2	73.5 ± 13.5	0.25
Height, cm, mean ± SD	160.6 ± 7.8	163.9 ± 8.6	0.25
Body mass index, kg/m^2^, mean ± SD	26.4 ± 3.6	27.2 ± 3.5	0.52

Systolic blood pressure, mmHg, mean ± SD	132 ± 19	134 ± 13	0.73
Diastolic blood pressure, mmHg, mean ± SD	79 ± 10	83 ± 8	0.21
Resting heart rate, bpm, mean ± SD	71 ± 11	71 ± 9	0.99

MMSE, total score, mean ± SD	26.7 ± 1.7	27.4 ± 1.3	0.19

ADAS-Cog, total score, mean ± SD	18.2 ± 4.4	18.7 ± 3.8	0.73

Abbreviations: MMSE, Mini-Mental State Examination; ADAS-Cog, Alzheimer's Disease Assessment Scale Cognitive.

Asterisk (*) indicates statistical significance for between-group comparisons usingt tests for normally distributed data the Wilcoxon Signed-Ranks Test for variables not following a normal distribution, and Chi-square test with Yates’s correction for categorical variables.

The changes in primary and secondary outcomes throughout the trial for each group are presented in Table 2. Dihydrogen-PQQ led to a significant increase in serum BDNF levels at the six-week follow up (*P* =  0.01); no alterations in BDNF levels were noted during the study in the placebo group (*P* =  0.27). Two-way ANOVA with repeated measures indicated a non-significant trend (treatment vs. time interaction) in the impact of interventions on BDNF levels, implying a tendency for dihydrogen-PQQ to upregulate BDNF levels compared to the placebo (*P* = 0.14). At the 6-week follow-up, irisin levels exhibited a trend towards an increase in the dihydrogen-PQQ group (*P* = 0.15) while showing a tendency to decrease in the placebo group (*P* = 0.08). Additionally, a non-significant trend for an interaction effect (time vs. intervention) concerning irisin levels was observed (*P* = 0.21), suggesting that dihydrogen-PQQ tended to upregulate irisin compared to the placebo.

**Table 2:** Clinical outcomes during the study. Values are mean ± SD.

	Experimental group (*n* = 18)		Control group (*n* = 16)		P*
	Baseline	Follow-up	Baseline	Follow-up	
*Serum biomarkers*					
BDNF (ng/mL)	20.9 ± 5.9	23.9 ± 7.2^†^	8.1 ± 2.7	8.7 ± 1.8	0.14
Irisin (ng/mL)	86.2 ± 49.8	99.2 ± 74.4	87.8 ± 53.4	67.9 ± 48.8	0.21

MMSE (total score)	26.7 ± 1.7	28.3 ± 1.7 ^†^	27.4 ± 1.3	29.3 ± 0.9 ^†^	0.58

*ADAS-Cog*					
Word recall (score)	2.5 ± 1.0	1.4 ± 1.1^†^	2.1 ± 1.0	1.4 ± 1.0^†^	0.21
Naming (score)	0.1 ± 0.2	0.1 ± 0.2	0.0 ± 0.0	0.0 ± 0.0	0.33
Commands (score)	0.4 ± 0.6	0.2 ± 0.4	0.3 ± 0.6	0.3 ± 0.4	0.26
Constructional praxis (score)	0.1 ± 0.2	0.1 ± 0.2	0.0 ± 0.0	0.1 ± 0.3	0.58
Ideational praxis (score)	0.0 ± 0.0	0.0 ± 0.0	0.3 ± 0.8	0.2 ± 0.5	0.77
Orientation (score)	0.3 ± 0.5	0.1 ± 0.2^†^	0.0 ± 0.0	0.1 ± 0.3	0.03
Word recognition (score)	11.5 ± 2.1	7.9 ± 4.5^†^	12.0 ± 0.0	9.1 ± 4.0^†^	0.78
Remembering test instructions (score)	0.9 ± 0.8	0.6 ± 0.6^†^	0.8 ± 0.7	0.4 ± 0.5^†^	0.70
Spoken language ability (score)	0.3 ± 0.6	0.4 ± 0.7	0.6 ± 0.8	0.6 ± 0.7	0.83
Word finding difficulty (score)	0.2 ± 0.4	0.2 ± 0.4	0.4 ± 0.7	0.4 ± 0.5	0.72
Comprehension (score)	0.8 ± 0.9	0.5 ± 0.6	1.2 ± 0.8	0.7 ± 0.6^†^	0.59
Concentration (score)	1.1 ± 0.9	0.7 ± 0.8^†^	1.1 ± 0.8	0.9 ± 0.8	0.36
Total (score)	18.2 ± 4.4	12.0 ± 6.7^†^	18.7 ± 3.8	14.2 ± 6.6^†^	0.42

*Safety panel*					
AST (IU/L)	24 ± 5	23 ± 5^†^	23 ± 3	23 ± 5	0.52
ALT (IU/L)	21 ± 8	20 ± 7	25 ± 9	24 ± 11	0.78
ALP (IU/L)	63 ± 17	68 ± 20^†^	77 ± 16	81 ± 18^†^	0.36
GGT (IU/L)	17 ± 4	17 ± 5	25 ± 20	25 ± 22	0.89
CK (IU/L)	96 ± 47	87 ± 44^†^	105 ± 50	111 ± 86	0.47
Total cholesterol (mmol/L)	6.2 ± 1.1	6.0 ± 0.9	6.9 ± 1.6	6.6 ± 1.5	0.74
LDL-cholesterol (mmol/L)	4.0 ± 0.9	3.7 ± 0.7^†^	4.4 ± 1.4	4.0 ± 0.9	0.17
HDL-cholesterol (mmol/L)	1.7 ± 0.3	1.7 ± 0.4	1.6 ± 0.4	1.5 ± 0.4	0.69
Triglycerides (mmol/L)	1.2 ± 0.4	1.2 ± 0.3	2.0 ± 1.4	1.8 ± 1.1	0.32
Glucose (mmol/L)	5.3 ± 0.4	5.4 ± 0.6	5.7 ± 0.7	5.5 ± 0.5 ^†^	< 0.01

Abbreviations: MMSE, Mini-Mental State Examination; ADAS-Cog, Alzheimer's Disease Assessment Scale Cognitive; BDNF, Brain-derived neurotrophic factor; AST, aspartate transaminase; ALT, alanine transaminase; ALP, alkaline phosphatase; GGT, gamma-glutamyl transferase; CK, creatine kinase; LDL, low-density lipoprotein; HDL, high-density lipoprotein.

Asterisk (*) indicates statistical significance for 2-way ANOVA interaction effect (time *vs*. treatment) or Friedman’s 2-way ANOVA by ranks. A dagger (†) indicates significant difference at *P* ≤  0.05 for within-group comparisons between baseline and follow-up.

Both the dihydrogen-PQQ mixture and the placebo significantly improved MMSE total scores compared to baseline values (*P* ≤ 0.05). Two-way ANOVA with repeated measures revealed no significant difference (treatment vs. time interaction) between interventions in influencing MMSE (*P* =  0.58). The dihydrogen-PQQ mixture resulted in a significant improvement in ADAS-Cog scores across five domains (word recall, orientation, word recognition, remembering test instructions, concentration) and total scores after 6 weeks of intake compared to baseline values (*P* ≤ 0.05). Similar results were observed in the control group, in which mean scores for word recall, word recognition, remembering comprehension, and total ADAS-Cog scores were significantly reduced at the 6-week follow-up (*P* ≤ 0.05). However, a significant interaction effect (time vs. treatment) was found for ADAS-Cog scores in the orientation domain (*P* = 0.03), with the dihydrogen-PQQ demonstrating superiority over placebo in improving this aspect of cognition.

The safety panel indices remained largely unaffected by either intervention. Still, serum concentrations of aspartate transaminase (AST), creatine kinase (CK), and LDL-cholesterol significantly decreased, while alkaline phosphatase (ALP) significantly increased after six weeks of dihydrogen-PQQ administration (*P* ≤ 0.05). In the control group, a significant rise in ALP levels accompanied by a reduction in glucose was identified (*P* ≤ 0.05). We found no significant interaction effect (time vs. treatment) between interventions across all safety biomarkers assessed during the trial (*P* > 0.05), except for a significant yet clinically irrelevant effect on blood glucose (*P* = 0.003).

**Fig. 1:**
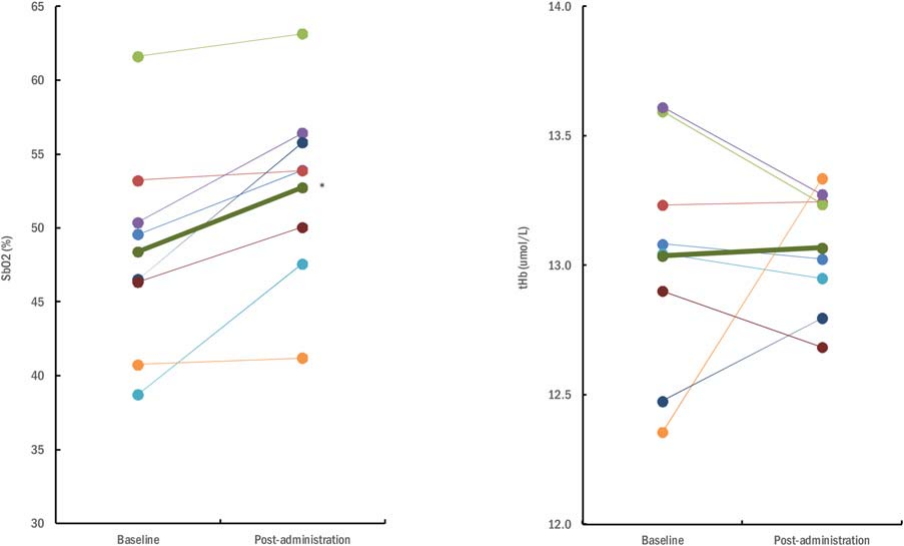
Individual changes in brain oxygenation indices (SbO_2_, cerebral oxygenation saturation; tHb, hemoglobin index) in participants receiving the experimental intervention. Thick line illustrates mean values. An asterisk (*) denotes statistical significance at *P* ≤  0.05 when comparing mean values between baseline and post-administration.

Changes in brain oxygenation indices in the prefrontal cortex for a subset of participants receiving the experimental intervention are illustrated in Fig. 1. SbO2 exhibited a significant increase during the trial, from 48.4 ± 7.2% at baseline to 52.8 ± 6.6% at 6-week post-administration (*P* = 0.005). No significant alterations in tHb levels were observed within the experimental group throughout the trial (13.0 ± 0.5 μmol/L at baseline vs. 13.1 ± 0.2 μmol/L at post-administration; *P* = 0.42).

Table 3 illustrates the alterations in brain metabolites observed among a subset of participants who underwent the experimental intervention. The experimental intervention resulted in a significant elevation of brain total choline (tCho) levels at two locations (left and right paracentral grey matter) during the trial (*P* ≤ 0.05); a strong trend for a non-significant increase was observed at the left frontal white matter, left parietal white matter, and right parietal mesial grey matter (*P* ≤ 0.20). Brain total creatine (tCr) exhibited overall resistance to the experimental intervention across all evaluated locations (*P* > 0.05), except for a significant reduction in tCr levels at the thalamus at follow-up (*P* = 0.01). Intriguingly, brain N-acetyl-aspartate (NAA) levels significantly increased at seven out of thirteen locations post-intervention (*P* ≤ 0.05), with a strong trend for a non-significant increase observed in remaining locations.

**Table 3:** Levels of brain metabolites during the trial in participants undergoing the experimental intervention. Values are mean ± SD.

	Baseline	Follow-up	P *
*Total choline (mM)*			
Thalamus	1.33 ± 0.18	1.36 ± 0.17	0.29
Left frontal white matter	1.90 ± 0.28	2.00 ± 0.36	0.10
Right frontal white matter	1.96 ± 0.27	1.86 ± 0.18	0.19
Left frontal grey matter	1.07 ± 0.27	1.07 ± 0.30	0.41
Right frontal grey matter	1.03 ± 0.23	1.06 ± 0.20	0.36
Left precentral white matter	1.72 ± 0.34	1.66 ± 0.14	0.25
Right precentral white matter	1.77 ± 0.38	1.74 ± 0.24	0.41
Left paracentral grey matter	1.32 ± 0.16	1.42 ± 0.27	0.04
Right paracentral grey matter	1.31 ± 0.31	1.45 ± 0.18	0.05
Left parietal white matter	1.67 ± 0.39	1.80 ± 0.31	0.08
Right parietal white matter	1.65 ± 0.24	1.70 ± 0.27	0.22
Left parietal mesial grey matter	0.92 ± 0.29	0.98 ± 0.30	0.23
Right parietal mesial grey matter	0.94 ± 0.19	1.00 ± 0.21	0.19

*Total creatine (mM)*			
Thalamus	6.27 ± 0.95	5.65 ± 0.48	0.01
Left frontal white matter	6.41 ± 0.64	6.40 ± 0.42	0.46
Right frontal white matter	6.48 ± 1.02	6.55 ± 0.75	0.31
Left frontal grey matter	5.35 ± 1.04	5.21 ± 0.91	0.24
Right frontal grey matter	5.65 ± 0.92	5.76 ± 0.77	0.26
Left precentral white matter	5.45 ± 0.58	5.46 ± 0.68	0.46
Right precentral white matter	5.82 ± 0.83	5.70 ± 0.64	0.26
Left paracentral grey matter	6.38 ± 1.21	6.37 ± 0.97	0.48
Right paracentral grey matter	6.49 ± 0.73	6.58 ± 0.54	0.13
Left parietal white matter	5.88 ± 0.60	5.67 ± 0.77	0.15
Right parietal white matter	6.04 ± 0.91	5.78 ± 0.79	0.10
Left parietal mesial grey matter	5.42 ± 1.53	5.55 ± 1.19	0.31
Right parietal mesial grey matter	5.72 ± 1.57	5.82 ± 1.37	0.33

*N-acetyl aspartate (mM)*			
Thalamus	8.43 ± 0.75	8.90 ± 0.85	0.01
Left frontal white matter	8.90 ± 0.96	9.64 ± 0.84	< 0.01
Right frontal white matter	8.61 ± 1.05	9.09 ± 1.21	0.01
Left frontal grey matter	4.94 ± 1.03	5.31 ± 1.15	0.04
Right frontal grey matter	5.24 ± 0.98	5.65 ± 1.10	0.02
Left precentral white matter	8.73 ± 1.23	8.95 ± 1.22	0.07
Right precentral white matter	8.95 ± 0.85	9.08 ± 0.76	0.11
Left paracentral grey matter	6.39 ± 1.13	6.51 ± 1.05	0.22
Right paracentral grey matter	6.36 ± 0.92	6.82 ± 0.81	0.02
Left parietal white matter	9.09 ± 0.95	9.30 ± 0.86	0.12
Right parietal white matter	9.23 ± 0.71	9.42 ± 0.75	0.06
Left parietal mesial grey matter	5.54 ± 1.11	6.01 ± 1.16	< 0.01
Right parietal mesial grey matter	5.90 ± 1.79	6.23 ± 1.61	0.07

Note: An asterisk (*) denotes statistical significance between baseline and follow-up values using *t* tests for normally distributed data and the Wilcoxon Signed-Ranks Test for matched data not following a normal distribution.

All participants who completed the trial did not report any significant side effects or adverse events from either intervention. A female participant, aged 70, reported a transient and mild episode of heartburn following the administration of the control intervention. Furthermore, two female participants, aged 71 and 75, reported that the control intervention had an excessively sweet taste. Following the experimental intervention, four female participants reported an improvement in well-being and a reduction in fatigue, along with enhanced energy levels and improved sleep. The compliance with the intervention was 96.9 ± 4.4% in the experimental group and 96.1 ± 4.9% in the control group, respectively (*P* = 0.30).

## 4 Discussion

Our RCT is likely the first to evaluate the concurrent administration of dihydrogen and PQQ in individuals aged 65 years and older with mild cognitive impairment. We observed that a six-week supplementation with dihydrogen and PQQ tended to upregulate mitochondria-related neurotrophic factors (BDNF and irisin), with serum BDNF levels increased significantly at six-week follow-up while no significant changes in BDNF levels were seen in the placebo group. Moreover, the combination outperformed the placebo in enhancing a specific cognitive domain (orientation) in elderly individuals with MCI. The dihydrogen-PQQ mixture also exhibited significant enhancements in cerebral oxygen saturation within the prefrontal cortex, elevated brain metabolites (NAA in particular) spanning both grey and white matter, and demonstrated the absence of side effects. The mixture shows promise as a safe and effective dietary intervention for enhancing mental orientation and brain metabolism in age-related mild cognitive decline.

A handful of preclinical and clinical studies have revealed the advantageous impact of dihydrogen on cognitive performance. The earlier study linking dihydrogen and cognition showed that hydrogen-rich saline ameliorated oxidative stress, cognitive impairment, and mortality in rats undergoing sepsis [11]. This was followed by several preclinical studies confirming the protective effects of hydrogen-rich saline against cognitive deficits induced by traumatic brain impairment [12], liver damage [13], amyloid β peptide deposition [14], methamphetamine-induced neurotoxicity [15], radiation [16], reoxygenation dysfunction [17], and paclitaxel-induced neuronal disorder [18]. A recent randomized controlled trial exhibited a tendency toward improved cognitive functioning in individuals aged 70 years and above who received hydrogen-rich water for six months [19]. Additionally, PQQ has shown pro-cognitive effects by itself in several animal studies, by preventing cognitive deficits induced by oxidative stress [20], the administration of noncompetitive N-methyl-D-aspartate (NMDA) receptor antagonist [21], D-galactose [22], and aging [23]. A randomized, double-blinded, placebo-controlled study demonstrated that 12-week supplementation with PQQ (20 mg per day) can prevent the reduction of brain function in elderly healthy subjects, particularly in attention and working memory [24]. Enhanced cognition scores in language domain were observed in healthy men and women aged 50–71 years subjected to a daily dose of 20 mg PQQ for 12 weeks [23]. Another study found that 12-week supplementation with PQQ disodium salt (12.5 mg/day) was effective in enhancing memory, attention, judgment, and cognitive function in middle-aged to elderly Japanese adults [25]. A recent study demonstrated improvements in composite memory and verbal memory in both younger and older adults (aged between 20 and 65 years) after supplementation with PQQ (20 mg per day) for 12 weeks [26]. Interestingly, a pilot trial involving fourteen middle-aged healthy adults, where both dihydrogen and PQQ were administered simultaneously for four weeks, revealed no significant effects on cognitive performance and biochemical indices [6]; the study served as an extension to a primary *in vitro* investigation but omitted to provide any specific numerical data for the human arm. The present study largely aligns with prior trials, affirming mild pro-cognitive effects of dihydrogen and PQQ. It broadens the scope to include a co-supplementation approach and specific supplementation period, offering a more comprehensive evaluation of brain metabolism and biomarkers of cellular senescence/mitochondrial function. Additionally, it includes elderly individuals with mild cognitive impairment.

We noted a significant elevation in circulating BDNF levels (primary outcome) after the dihydrogen-PQQ supplementation compared to the baseline levels, indicating an average increase of 14.4%. A non-significant trend for an interaction effect was observed in the levels of BDNF (also irisin), suggesting that the dihydrogen-PQQ mixture may have a modest advantage over the placebo in boosting both biomarkers. BDNF and irisin are acknowledged as neurotrophic and neuroprotective factors [28], and either dihydrogen, PQQ, or both may upregulate these biomolecules through various mechanisms, including modulation of neuronal growth factors, neurotransmission, reelin signaling, and brain mitochondrial bioenergetics. A recent study by Bloomer et al. [6] revealed no significant effects of the dihydrogen-PQQ mixture on cognition and serum indicators of mitochondrial function (BDNF, FGF-21, irisin) in healthy middle-aged adults. However, our study observed a significant impact of the mixture on mental orientation and mitochondrial function. This discrepancy suggests that the possible beneficial effects of dihydrogen and PQQ may be more pronounced in subjects with early-stage memory loss or other cognitive disabilities, such as MCI. Interestingly, our findings revealed a substantial disparity in baseline BDNF levels between the control group (8.1 ng/mL) and the experimental group (20.9 ng/mL). This discrepancy suggests potential heterogeneity in neurogenesis between the groups, with lower baseline levels possibly indicative of diminished neuronal development, survival, and synaptic plasticity. To comprehensively assess the impact of dihydrogen and PQQ on BDNF levels, future studies should aim to recruit subjects with comparable baseline serum BDNF concentrations. We also observed that dihydrogen and PQQ significantly surpassed placebo in improving ADAS-Cog scores for orientation, demonstrating an average improvement of 22.2% in the orientation score following supplementation with the dihydrogen-PQQ mixture. The enhanced cognitive orientation observed in our trial may be attributed to various biological pathways activated by the co-administration of dihydrogen and PQQ. Both compounds are recognized for their antioxidant activity, potentially mitigating oxidative stress associated with aging and cognitive decline [27]. The cognitive improvement linked to the mixture could also be due to the promotion of mitochondrial and neuronal biogenesis, as suggested by a recent *in vitro* study [6].

Our study unveiled a potential influence of dihydrogen and PQQ on cerebral oxygenation within the prefrontal cortex. Remarkably, the combination significantly enhanced brain oxygenation saturation by an average of 9.5%, with no discernible effect on hemoglobin index. The medial prefrontal cortex plays a crucial role in mental orientation in space, time, and person [29], and dihydrogen and PQQ may enhance blood flow and provide additional oxygen to this specific brain network. Our findings align with preliminary studies that demonstrated the independent effects of PQQ or dihydrogen on cerebral blood flow and oxygen metabolism in the prefrontal cortex [30,31]. The concurrent administration of both components could potentially augment cerebral oxygenation, associated with improved function in a brain region governing higher-order (executive) cognitive processes. A strong effect of dihydrogen and PQQ was also demonstrated for enhancing brain metabolism, in which the dihydrogen-PQQ mixture increased NAA levels across a majority of locations evaluated in the present trial. The highest mean increase was found at left parietal mesial grey matter (8.6%), followed by left frontal white matter (8.3%), and right frontal grey matter (7.9%). NAA is synthesized within neuronal mitochondria, and is acknowledged as a crucial biomarker of neuronal viability and density [32]. Elevated NAA levels have been positively linked to working memory performance [33] whereas decreased levels have been observed in individuals with MCI and dementia [34]. Our findings suggest that the enhanced cognition in the elderly with MCI may be attributed to this 'pro-metabolic' effect of the mixture, particularly targeting NAA in the frontal cortex. Elevated NAA concentrations may indicate an improved tissue environment resulting from the intervention, suggesting that the dihydrogen-PQQ mixture could potentially reverse neuronal/axonal loss or address compromised neuronal metabolism. In addition, the mixture augmented the brain levels of tCho, a biomarker of neuronal membrane turnover. While interpreting changes in tCho is complex due to various factors influencing the observed tCho resonance [35], an increase in tCho may indicate an elevated rate of membrane synthesis induced by the dihydrogen-PQQ mixture. This effect is evident in the paracentral grey matter, a functional area associated with motor and sensory functions of the lower limbs; whether the increase in tCho induced by the dihydrogen-PQQ mixture translates into functional benefits is yet to be investigated. These effects might be particularly pronounced in individuals experiencing stress, including sleep deprivation, physical exercise, and age-related conditions. However, the specific individual contributions of PQQ and dihydrogen remain unknown and should be the focus of future research. These components might elicit similar effects through distinct mechanisms; investigating whether their effects are additive or synergistic warrants further investigation.

While our study design was robust, there are several limitations in our trial. We specifically focused on a sample of elderly with borderline cognitive impairment, and whether the dihydrogen-PQQ mixture produces similar effects in other populations with moderate-to-severe cognition decline, including AD and other dementias, remains unknown and warrants further investigation. While our sample size aligned with the power analysis for the primary outcome (change in serum BDNF levels from baseline to the 6-week follow-up), the number of participants may be deemed insufficient for interpreting all clinical parameters. In addition, the constrained sample size (with a predominantly female population analyzed in our study) limits the ability to discern potential gender-specific associations. The multi-nutrient formulation of the mixture complicates pinpointing the individual contribution of dihydrogen and PQQ to the observed effects. Furthermore, our assessment spanned six weeks, and extending the administration period may yield different outcomes. Finally, a more comprehensive mechanistic approach, involving a broader array of biomarkers and more frequent sampling, might be necessary to unravel the molecular pathways influenced by dihydrogen and PQQ in the brain (and other organs) of individuals with MCI.

## 5 Conclusion

The six-week supplementation of dihydrogen and PQQ appears to mildly mitigate cognitive decline in elderly individuals with mild cognitive impairment, possibly due to its positive impact on mitochondria-related neurotrophic factors, cerebral hemodynamics, and brain metabolism. Recommending this mixture could be important in addressing the prevalent issue of mild cognitive impairment in the elderly. However, additional research is imperative to validate these findings across diverse populations with cognitive decline.

## Statement of ethics

The ethical approval was granted by the local IRB at the University of Novi Sad (#A13-CH-2023). Consent to Participate Statement

The informed written consent was obtained from all respondents to participate in the study. The research was conducted ethically in accordance with the World Medical Association Declaration of Helsinki.

## Conflict of interests

SB, DN, NT, MR, DK, JC, JO, vs and SMO declare there are no competing interests. TWL reports consulting fees from CalerieLife, and JT is employed by CalerieLife, the company that supplied the supplements examined in this study.

## Funding information

This study was partially funded by the Provincial Secretariat for Higher Education and Scientific Research (142-451-2597/2021-01).

## Data availability statement

All the data analyzed for this study are available within the article. For additional inquiries, please contact the corresponding author.
